# MLLT11 Regulates Endometrial Stroma Cell Adhesion, Proliferation and Survival in Ectopic Lesions of Women with Advanced Endometriosis

**DOI:** 10.3390/ijms25010439

**Published:** 2023-12-28

**Authors:** Katharina Proestling, Heinrich Husslein, Quanah James Hudson, Matthias Witzmann-Stern, Barbara Widmar, Zsuzsanna Bagó-Horváth, Lejla Sandrieser, Alexandra Perricos, René Wenzl, Iveta Yotova

**Affiliations:** 1 Department of Obstetrics and Gynecology, Medical University of Vienna, Waehringer Guertel 18-20, A-1090 Vienna, Austria; katharina.proestling@meduniwien.ac.at (K.P.); heinrich.husslein@meduniwien.ac.at (H.H.); quanah.hudson@meduniwien.ac.at (Q.J.H.); matthias.witzmann-stern@meduniwien.ac.at (M.W.-S.); barbara.widmar@meduniwien.ac.at (B.W.); lejla.sandrieser@meduniwien.ac.at (L.S.); alexandra.perricos@meduniwien.ac.at (A.P.); rene.wenzl@meduniwien.ac.at (R.W.); 2 Department of Pathology, Medical University of Vienna, Waehringer Guertel 18-20, A-1090 Vienna, Austria; zsuzsanna.horvath@meduniwien.ac.at

**Keywords:** MLLT11, endometriosis, primary endometrial stroma cells, proliferation, cell cycle, adhesion, apoptosis resistance, apoptosis

## Abstract

*MLLT11* is a gene implicated in cell differentiation and the development and progression of human cancers, but whose role in the pathogenesis of endometriosis is still unknown. Using quantitative RT-PCR and immunohistochemistry, we analyzed 37 women with and 33 women without endometriosis for differences in MLLT11 expression. We found that MLLT11 is reduced in the ectopic stroma cells of women with advanced stage endometriosis compared to women without endometriosis. *MLLT11* knockdown in control stroma cells resulted in the downregulation of their proliferation accompanied by G1 cell arrest and an increase in the expression of p21 and p27. Furthermore, the knockdown of *MLLT11* was associated with increased apoptosis resistance to camptothecin associated with changes in BCL2/BAX signaling. Finally, *MLLT11* siRNA knockdown in the control primary stroma cells led to an increase in cell adhesion associated with the transcriptional activation of ACTA2 and TGFB2. We found that the cellular phenotype of *MLLT11* knockdown cells resembled the phenotype of the primary endometriosis stroma cells of the lesion, where the levels of MLLT11 are significantly reduced compared to the eutopic stroma cells of women without the disease. Overall, our results indicate that MLLT11 may be a new clinically relevant player in the pathogenesis of endometriosis.

## 1. Introduction

Endometriosis is an estrogen-driven gynecological condition affecting up to 10% of the women of a reproductive age [1]. It is characterized by the formation of endometriosis lesions outside the uterus, predominantly in the peritoneal cavity. These ectopic growths contain endometrial glands and stromal cells resembling endometrium [2]. It is a chronic inflammatory disease, commonly resulting in symptoms including chronic pelvic pain, dysmenorrhea, dyspareunia and fatigue and is often associated with infertility, strongly affecting the quality of life of affected women [2]. Currently, peritoneal endometriotic lesions are mainly diagnosed using laparoscopy, as no clinically approved diagnostic biomarkers exist. The nonspecific nature of the symptoms and the lack of reliable diagnostic markers, together with uncertain disease etiology, often leads to a significant delay in the diagnosis and treatment of patients with the disease [3]. Although the etiology of the disease remains unclear, the retrograde transport of menstrual endometrial debris into the peritoneal cavity is the most widely accepted hypothesis for the origin of ectopic lesions [4]. Endometriosis is a benign disease, which shows progressive and invasive growth, a high recurrence rate and tends to spread [5,6]. In order for endometriotic lesions to develop, endometrial cells in the peritoneal cavity must adhere, proliferate, invade and differentiate while avoiding or coopting the immune system [7,8]. However, the growth of endometriotic lesions is tightly controlled, while the uncontrolled proliferation of ectopic lesions is rarely observed in endometriosis [9].

Nevertheless, a range of studies have shown that genes with an altered expression in a diversity of cancers are also deregulated in endometriotic tissue [10] and may contribute to the pathogenesis of endometriosis. For example, patients with endometriosis have a higher risk of developing some types of ovarian cancer [11]. Previous reports have shown that shared alterations in the expression levels of certain genes in both endometriotic lesions and endometriosis-associated ovarian carcinomas exist [12]. This indicates that examining deregulated genes in both diseases may help understand the pathogenesis of endometriosis and to define the molecular basis for the differences between these benign and malignant conditions.

Mixed-lineage leukemia translocated to 11 (*MLLT11*), also called the ALL1-fused gene from the chromosome 1q (*AF1Q*) gene, has been found to be overexpressed in ovarian cancer and to be associated with a poor patient outcome [13]. The gene encodes a small 9 kDa protein originally defined as an oncogenic factor associated with a genetic translocation (t (1;11) (q21;q23)) present in certain types of leukemia [14,15]. The gene functions as an oncogene by regulating the epithelial to mesenchymal transition of ovarian cells to ensure their high plasticity, increased invasiveness and motility [13]. In ovarian cancer cells, the overexpression of MLLT11 was also associated with an activation of WNT/beta-catenin/S100A4 oncogenic signals, resulting in malignant tumor progression and metastasis formation and reduced sensitivity to cancer therapy [13]. On the other hand, MLLT11 has also been identified as mediator of basal and 4-HPR-induced apoptosis in ovarian cancer cells, illustrating the somewhat contradictory dual proapoptotic and protumorigenic behavior of the gene in ovarian cancer cells [16]. A functional role of MLLT11 in tumor progression was also reported in human breast [17] and endometrial carcinomas [18], as well as in a number of other human solid tumors [19,20]. In contrast to this oncogenic role, in the nervous system, *MLLT11* functions as a tumor suppressor, with a high level of expression in normal brain tissues compared with decreased expression correlating with the malignant tumor grade [21]. 

Despite the considerable advances made in understanding the role of MLLT11 in human carcinogenesis and in cortical projection neuron morphogenesis during development [22], the role of this gene in the pathogenesis of endometriosis remains unclear. Therefore, in this study, we analyzed the differences in the expression levels of MLLT11 in women with and without endometriosis and evaluated the role of this gene in disease pathogenesis using primary endometrial stroma cells. 

## 2. Results

### 2.1. MLLT11 Is Downregulated in Ectopic Lesions of Women with Stage III and IV Endometriosis

**Figure 1:**
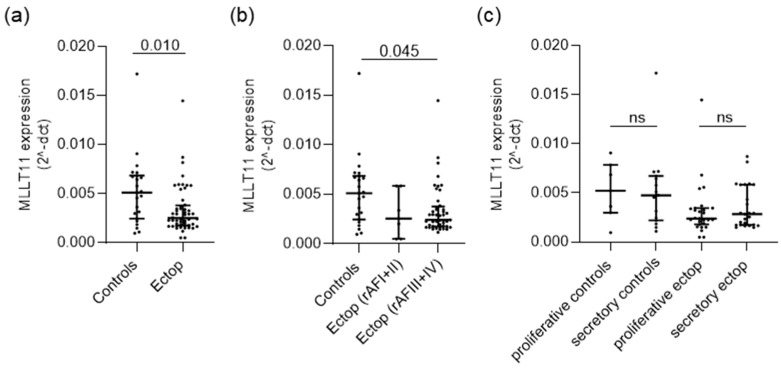
*MLLT11* mRNA expression is reduced in endometriosis lesions. (**a**) Gene expression of *MLLT11* is significantly decreased in ectopic lesions (n = 52) compared to the eutopic endometrium of women without endometriosis (n = 23). (**b**) Compared to controls, ectopic MLLT11 expression does not significantly change in mild disease (rAF I + II, n = 7) but shows a significant decrease in more severe endometriosis (rAF III + IV, n = 45). Disease stages according to revised American Fertility Society (rAF) scores. (**c**) *MLLT11* expression does not significantly differ between the proliferative (n = 7) and secretory (n = 15) stages of the menstrual cycle in control eutopic endometrium or ectopic lesions (proliferative n = 27, secretory n = 25). *MLLT11* gene expression in (**a**–**c**) was normalized to ACTB, and data are presented as scatter dot plots including the median relative expression levels and interquartile ranges for each group. Data were analyzed using Mann–Whitney test (**a**) or Kruskal–Wallis test adjusted for multiple testing using Dunn’s multiple comparisons test (**b**,**c**). Adjusted p values (adjp) < 0.05 were considered significant, with nonsignificant differences indicated by ns. Controls: endometrial tissue of women without endometriosis, Ectop: tissues from endometriosis lesions.

To identify changes that may occur in *MLLT11* expression levels in endometriosis, we compared the control endometrium of women without endometriosis to ectopic lesions using qRT-PCR. This included 23 women without and 31 women with endometriosis—with some patients contributing more than one lesion sample (Supplementary Table S1A). This showed that *MLLT11* expression is downregulated (0.49 median fold change, adjp value = 0.010) in ectopic lesions of women with endometriosis compared to the eutopic endometrium of women without the disease (Figure 1a). Women with severe stage endometriosis (the revised American Fertility Society (rAFS) score III + IV) showed significantly lower levels of *MLLT1* expression compared to women without the disease (0.53 median fold change, adjp value = 0.045, Figure 1b). However, it cannot be excluded that MLLT11 expression is also reduced in rAFS I + II stage ectopic tissue, given that a relatively small number of samples of this type of tissue were examined (n = 7). *MLLT11* expression did not significantly vary during the menstrual cycle in either the control or ectopic tissue (Figure 1c). 

**Figure 2:**
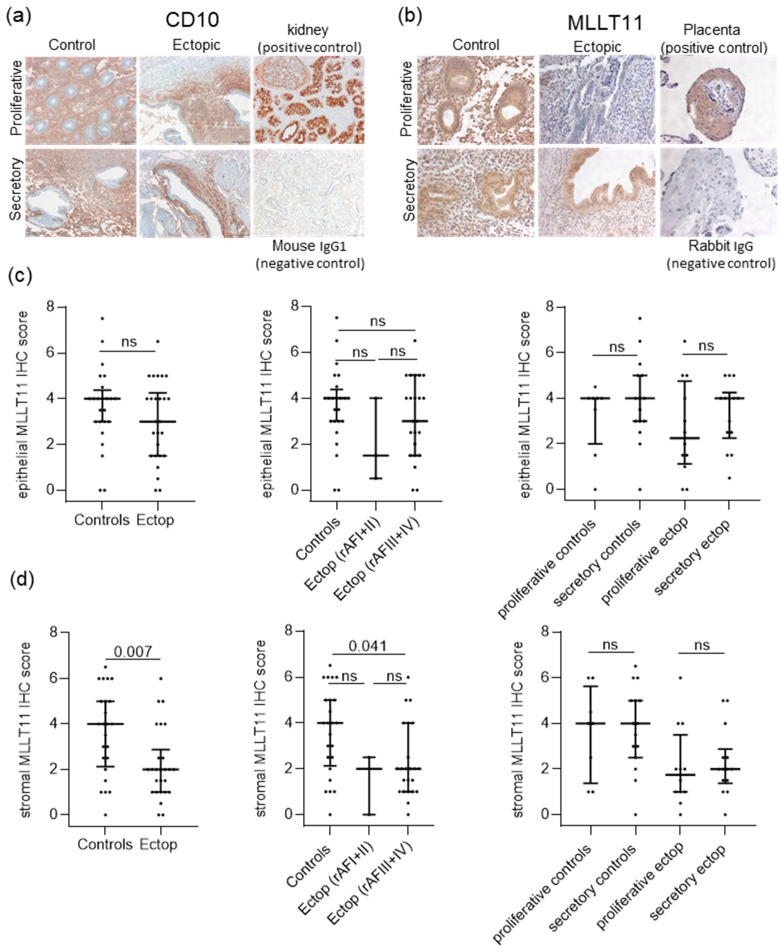
Immunohistochemical analyses of MLLT11 expression levels in ectopic endometriosis lesions (n = 26) and in control eutopic endometrium from women without endometriosis (n = 24). (**a**) Representative pictures of CD10 staining in tissues of control and ectopic endometrium in the proliferative and secretory menstrual cycle phases are shown at a magnification of 200×. (**b**) Representative pictures of MLLT11 staining in controls and ectopic endometrium in the proliferative and secretory menstrual cycle phases. (**c**,**d**) The intensity (0–3) and the percentage (0–3) of the stained cells were assessed and combined to define the final IHC score (0–9). For statistical evaluation of the protein MLLT11 score, (**c**) epithelial and (**d**) stromal immunostainings were analyzed separately at magnification of 200×. (**c**,**d**, middle) MLLT11 protein expression in mild (rAF I + II, n = 3) and more severe (rAF III + IV, n = 23) disease stages (revised American Fertility Society (rAF) score. (**c**,**d**, right) MLLT11 protein expression does not significantly differ between the proliferative and secretory stages of the menstrual cycle in control endometrium (n = 8 and 15, respectively) or ectopic lesions (n = 12 and 14, respectively). All results (**c**,**d**) are expressed as scatter dot plots including the median relative expression levels and interquartile ranges in each group. Data were analyzed using Mann–Whitney tests (**c**,**d** left) or Kruskal–Wallis test adjusted for multiple testing using Dunn’s multiple comparisons test (**c**,**d** middle and right). Adjusted *p* values (adjp) < 0.05 were considered significant. The nonsignificant differences are indicated by ns. Control: endometrial tissue of women without endometriosis, Ectop: tissues from endometriosis lesions.

Next, we used immunohistochemistry to localize MLLT11 protein expression in the endometrial tissue. As paraffin-embedded tissue was not available for all samples included in the mRNA expression analysis cohort, we had to collect additional samples. Therefore, the sample cohort used for the IHC analysis had only a 40.4% overlap with the sample cohort used for the mRNA expression analysis and, in the end, included 24 women without and 19 women with endometriosis—with some patients contributing more than one lesion sample (Supplementary Figure S1a and Table S1b). In addition, to be able to define the endometrial stroma cell population within the lesions, we stained all ectopic tissue samples with CD10, a verified endometrial stroma marker [23] (Figure 2a).

Our IHC analysis showed that MLLT11 is expressed in both stromal and epithelial cells but appeared to show differences between these two compartments (Figure 2b). The quantification of staining showed that the levels of MLLT11 in glandular epithelial cells did not differ between eutopic control endometrium and endometriotic lesions (Figure 2c, left). When we stratified the analysis according to the endometriosis disease stage and menstrual cycle phase, we also did not see differences in the levels of glandular epithelial cell MLLT11 expression between the groups (Figure 2c, middle and right). 

In contrast, in the stromal cell compartment, MLLT11 positive cells were significantly reduced in the ectopic lesions (0.5 median fold change, adjp value = 0.007) when compared to the eutopic control endometrium (Figure 2d, left). This indicates that the MLLT11 reduction seen in the ectopic lesions at the RNA level is due to reduced gene expression in the stromal cells (Figure 1a). When patients were classified according to their disease stage, a significant reduction in ectopic stromal cell MLLT11 expression was only seen in the advanced rAFS III +IV stages of endometriosis (0.5 median fold change, adjp value = 0.041) (Figure 2d, middle). However, it cannot be excluded that MMLT11 expression is also reduced in rAFS I + II stage ectopic stroma, given the small number of samples of this type of tissue (n = 3). There was no significant difference in MLLT11 stromal endometrial expression between the proliferative and secretory phases of the menstrual cycle for any tissue type (Figure 2d, right). 

### 2.2. MLLT11 Knockdown Impairs Cells Proliferation in Primary Endometrial Stromal Cells of Women without Endometriosis

To gain insight into the biological role of reduced MLLT11 expression in endometriosis lesions, we carried out *MLLT11* siRNA knockdown experiments in primary endometrial stroma cells (hESCs) derived from the endometrial tissue of four patients without endometriosis. 

**Figure 3:**
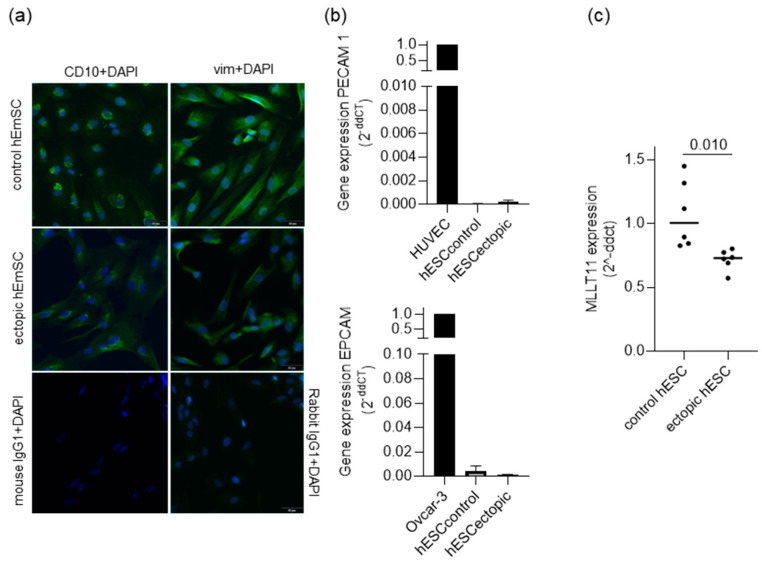
Characterization of primary stromal cell cultures. (**a**) Representative pictures of CD10 (left) and vimentin (right) immunofluorescence staining in primary endometrial stromal cells derived from control eutopic endometrium and ectopic endometriosis lesions. (**b**) RT-qPCR analysis shows that primary stromal cells derived from control eutopic endometrium (n = 4) and ectopic endometriosis lesion (n = 4) have very low levels of endothelial and epithelial markers *PECAM1* (top) and *EPCAM* (bottom), indicating their purity. HUVEC and OVCAR-3 were used as positive controls for endothelial and epithelial cells, respectively. Data are presented as bar graphs, and positive controls were set to 1. (**c**) RT-qPCR analysis of *MLLT11* expression in primary stroma cells derived from control eutopic endometrium (control hESCs, n = 6) and ectopic endometriosis lesions (ectopic hESCs, n = 6). *MLLT11* gene expression was significantly lower in ectopic hESCs compared to control hESCs. Data are presented as a scatter blot including the median expression level of each group and analyzed using *t*-test. The patient characteristics of the women from whom the primary cells were derived from are listed in Supplementary Table S1C.

To test if this in vitro system accurately represents the situation in patient lesions, we first characterized the purity of our primary cell cultures. All primary cultures derived from either control eutopic endometrium and endometriosis lesions were positive for endometrial stroma cell markers CD10 and vimentin (Figure 3a) and negative for endothelial (*PECAM1*) and epithelial (*EPCAM*) markers (Figure 3b), indicating they represent the endometrial stromal cell compartment. 

**Figure 4:**
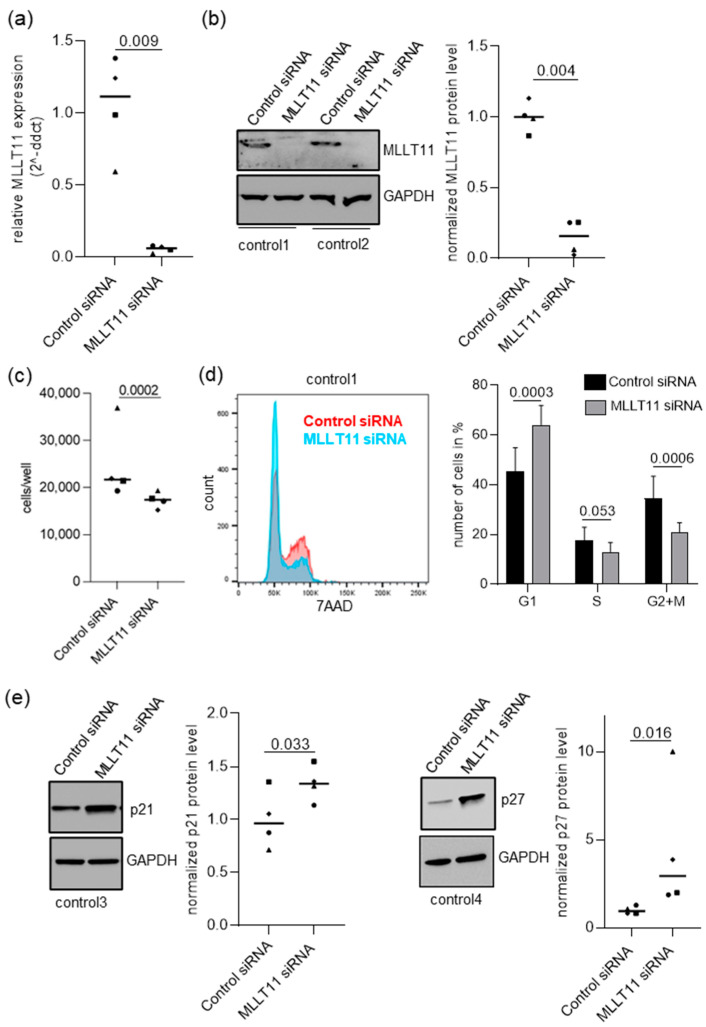
*MLLT11* knockdown leads to an increase in cells in the G1 phase and a decrease in cells in the S and M+G2 phases associated with an increase in p21 and p27 levels. (**a**) MLLT11 gene and (**b**) protein expression after *MLLT11* siRNA knockdown experiments in primary stroma cells obtained from endometrial tissue of four women without the disease. (**c**) Reduced proliferation rate in *MLLT11* siRNA knockdown cells (n = 4) compared to control siRNA-treated cells (n = 4). (**d**) *MLLT11* siRNA knockdown leads to an increase in cells in the G1 phase and a decrease in cells in the S and G2 + M phases. (Left): Representative DNA profiles obtained from flow cytometry analysis of PI stained primary stromal cells 72 h after transfection with *MLLT11* siRNA or control siRNA. (Right): The percentage of cells in each cell cycle phase is plotted as mean + SD of primary stromal cells obtained from endometrial tissue of four different women without the disease. Data were analyzed using multiple *t*-test. (**e**) The p21 and p27 protein are significantly upregulated following *MLLT11* knockdown. Representative examples of Western blot analysis following *MLLT11* knockdown of p21 and p27, together with the GAPDH loading control. Densitometry of p21 and p27 protein levels from Western blots were normalized to the loading control. In (**a**–**c**,**e**), the results are expressed as scatter dot plots including the median relative expression levels in each group. Symbols—○: for control1, □: for control2, Δ: for control3, ◊: for control4. Data were analyzed using paired *t*-test (**a**–**c**) and Wilcoxon matched-pairs signed rank test (**e**). The *p* values < 0.05 were considered as significant.

Secondly, we conducted an RT-qPCR analysis of *MLLT11* expression in primary stroma cells derived from six control endometrial samples and six ectopic lesion samples of women with endometriosis (Figure 3c). The results mirrored what was seen in vivo, with a significant reduction in *MLLT11* expression in ectopic compared to control hESCs (0.27 median fold change, *p* value = 0.010) (Figure 3c). Therefore, we proceeded with the *MLLT11* knockdown experiments in four out of six control primary stroma cell lines. We were able to achieve efficient knockdown, with *MLLT11* mRNA reduced to a mean of 5.4% (*p* = 0.009) of the control knockdown cells (Figure 4a). This resulted in a reduction in MLLT11 protein levels to a mean of 15.7% (*p* = 0.004) of the control knockdown cells (Figure 4b).

Next, we addressed the consequences of this reduction in MLLT11 levels on primary endometrial stroma cell biology. First, we assessed cell proliferation in these cells following *MLLT11* knockdown. We found that cell proliferation was significantly reduced by a median of 19.8% (*p* = 0.0002) compared to the control knockdown cells (Figure 4c). Additionally, the knockdown of *MLLT11* in the control primary hESCs resembled the differences in the proliferation of the control and ectopic primary hESCs (EcESCs), showing reduced proliferation of EcESCs compared to the controls by a median of 13.6% (*p* = 0.031, Supplementary Figure S1b).

The knockdown of *MLLT11* was not associated with any change in cell survival, with no significant difference in apoptosis detected using a 7AAD/AnnexinV flow cytometry assay (Supplementary Figure S2a). A decrease in the BCL2/BAX ratio is another indication of apoptosis [24], and this did also not significantly differ between the *MLLT11* knockdown and control siRNA-transfected cells (Supplementary Figure S2b). However, *MLLT11* knockdown did affect the cell cycle, with a 7AAD flow cytometry assay showing a significant increase in the number of cells in the G1 phase (141.0%, adjp = 0.0003) and a significant decrease in the number of cells in the S phase (73.9%, adjp = 0.053) and G2/M phases (60.8%, adjp = 0.0006) of the cell cycle (Figure 4d). This was associated with the significant upregulation of the levels of expression of the cell cycle checkpoint regulatory proteins p21 (1.39-fold, *p* = 0.033) and p27 (3.11-fold, *p* value = 0.016) (Figure 4e). The increased levels of p27 protein under MLLT11 knockdown was not associated with any significant changes in CDKN1B transcription (Supplementary Figure S1c). Therefore, MLLT11 may act as negative post-transcriptional regulator of the gene in control hESCs. Overall, the G1 arrest observed in these experiments may explain the effect of *MLLT11* knockdown on proliferation.

### 2.3. MLLT11 Mediates Camptothecin-Induced Apoptosis in Primary Endometrial Stroma Cells

**Figure 5:**
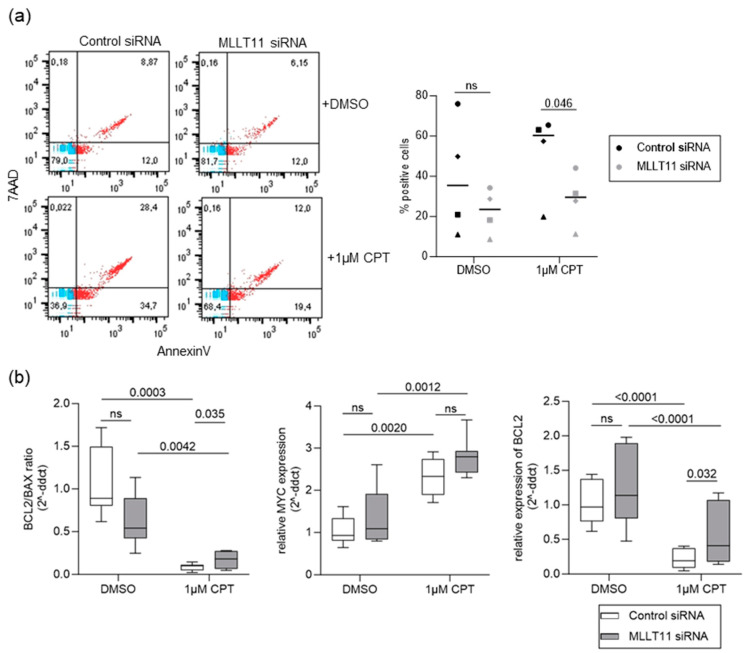
(**a**) Decreased number of apoptotic cells is seen in 24 h camptothecin (CPT)-treated cells 72 h after *MLLT11* siRNA knockdown. (Left): Representative flow cytometry scatter plots for AnnexinV+ versus 7-AAD+cells. Primary stromal cells obtained from endometrial tissue of four women without endometriosis were treated for 24 h either with 0.1% DMSO or with 1 µM CPT, together with either control siRNA or *MLLT11* siRNA for 72 h. (Right): No significant change in the number of apoptotic cells (AnnexinV+/7AAD (early apoptotic) plus AnnexinV+/7AAD+ (late apoptotic)) was observed between *MLLT11* knockdown and control siRNA-transfected cells in DMSO-treated cells. In CPT-treated cells, the number of total apoptotic cells was significantly decreased after *MLLT11* knockdown. The results are expressed as scatter dot plots including the median relative expression levels and interquartile ranges in each group. Symbols—○: for control1, □: for control2, Δ: for control3, ◊: for control4. Data were analyzed using paired *t*-test adjusted by Bonferroni correction, with *p* values < 0.05 considered as significant. (**b**) (Left), BCL2/BAX ratio (middle), relative cMYC expression (right), relative BCL2 expression in 24 h CPT-treated cells 72 h after *MLLT11* siRNA knockdown. Primary stromal cells obtained from eutopic endometrium of women without endometriosis were treated for 24 h either with 0.1% DMSO or with 1 µM CPT, together with either control siRNA or *MLLT11* siRNA for 72 h. Results are expressed as box plots including Tukey whiskers. Data were analyzed using Sidak’s multiple comparison tests from three biological replicates.

It has been shown that MLLT11 overexpression increases the sensitivity of human squamous cell carcinoma A431 parent (AP) cells and ovarian cancer cell lines to apoptotic drug stimulation [16,25]. Therefore, we tested whether reduced MLLT11 expression in endometriosis lesions is associated with increased resistance to apoptotic stimuli. To do this, we added 1 µM of the apoptosis-inducing agent camptothecin (CPT) to the medium 48 h after *MLLT11* or control knockdown and incubated for another 24 h before assessing the effect on apoptosis. Flow cytometry analysis of AnnexinV/7AAD positive cells to assess the number apoptotic cells showed no difference in the DMSO-treated cells (Figure 5a), consistent with an earlier experiment (Supplementary Figure S2a). However, while CPT treatment led to an increase in apoptotic cells in the control knockdown cells, apoptosis was not increased in the *MLLT11* knockdown cells, with 51.0% less apoptosis in *MLLT11* siRNA than in the control treated cells (adjp = 0.046) (Figure 5a, right). This effect was associated with a significant increase in the *BCL2/BAX* ratio in the knockdown CPT-treated cells (0.810 median fold change, adjp = 0.035) compared to the control siRNA CPT-treated cells (Figure 5b left). These results indicate that MLLT11 is involved in the regulation of CPT-induced apoptosis in primary endometrial stromal cells.

CTP-induced apoptosis in hESCs was associated with a significant upregulation in the levels of *c-MYC* expression (1.51 median fold change, adjp = 0.0020 for control siRNA-treated cells and 1.57 median fold change, adjp = 0.0012 for MLLT11 siRNA-treated cells (Figure 5b, middle)), accompanied by downregulation of the level of *BCL2* expression (0.802 median fold change, adjp < 0.0001 for control siRNA-treated cells and 0.643 median fold change, adjp < 0.0001 for MLLT11 siRNA-treated cells (Figure 5b right)) and a decreased *BCL2/BAX* ratio (0.887 median fold change, adjp = 0.0003 for control siRNA-treated cells and 0.664 median fold change, adjp = 0.0042 for MLLT11 siRNA-treated cells (Figure 5b, left)) with or without MLLT11 knockdown. However, MLLT11 knockdown was associated with significant differences in the degree of *BCL2* transcriptional deregulation under CTP stimulation irrespective of the *c-MYC* levels. The *BCL2* expression was significantly lower in control siRNA-transfected cells compared to MLLT11 knockdown cells (0.528 median fold change, adjp = 0.032). Overall, these results indicate that MLLT11 regulates the sensitivity of hESCs to CPT-induced apoptosis via the transcriptional regulation of *BCL2* expression.

### 2.4. MLLT11 Suppresses Adhesion in Primary Endometrial Stroma Cells of Women without Endometriosis

**Figure 6:**
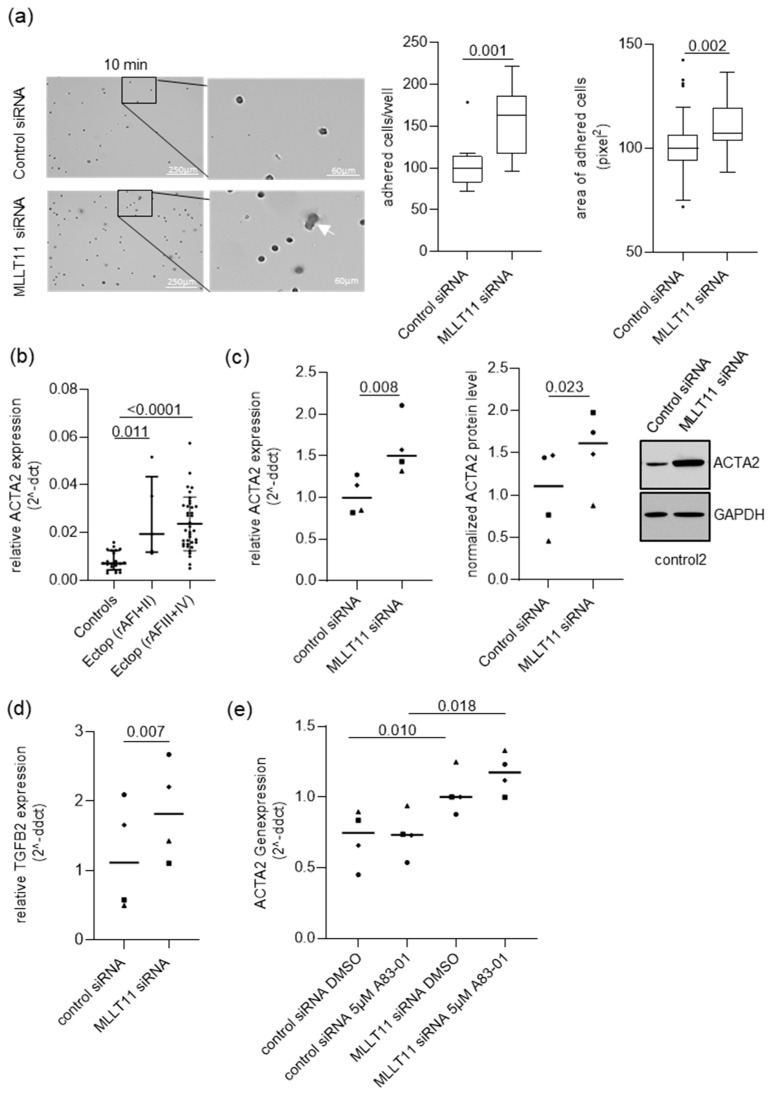
Number of adhering cells and area of adhesion are increased following *MLLT11* knockdown. (**a**) (Left): Representative images of control and knockdown cells at 100× and 400× magnification of the indicated magnified area stained with Chrystal Violet. The arrow shows a cell with increased adhesion area. (Middle): Quantification of the number of adhered cells to fibronectin- and collagen-coated plates after 72 h of *MLLT11* or control siRNA treatment stained 10 min after seeding. (Right): Quantification of the area of adhered cells to fibronectin- and collagen-coated plates after 72 h of *MLLT11* or control siRNA knockdown stained 10 min after seeding. Four independent image fields from three technical replicates in primary cells of four women were counted using ImageJ software V1.8.0. Results are expressed as boxplots with Tukey-based whiskers, and *p* values were calculated using the Wilcoxon matched-pairs signed rank test. (**b**) Gene expression of *ACTA2* is significantly increased in the ectopic lesions of women with mild (rAF I + II, n = 5) and high and more severe (rAF III + IV, n = 38) endometriosis (revised American Fertility Society score) compared to the eutopic endometrium of women without endometriosis (n = 23). Gene expression of *ACTA2* was normalized to ACTB and data in (**b**) are presented as scatter dot plots including the median relative expression levels and interquartile ranges in each group. Data were analyzed using Mann–Whitney test adjusted using Bonferroni. (**c**) Altered gene expression and protein levels of ACTA2 after *MLLT11* siRNA knockdown in primary stroma cells obtained from endometrial tissue of four women without the disease. Representative examples of Western blot analysis following *MLLT11* knockdown of ACTA2, together with the GAPDH loading control. Densitometric analysis of ACTA2 levels from Western blots normalized to the loading control. (**d**) Altered gene expression levels of *TGFB2* after *MLLT11* siRNA knockdown in primary stroma cells obtained from endometrial tissue of four women without the disease. (**e**) The 24 h inhibition of the TGFB signaling by 5 µM of A83-01 24 h after *MLLT11* siRNA knockdown in primary stroma cells obtained from endometrial tissue of four women without the disease. Gene expression of *ACTA2* and *TGFB2* in (**c**–**e**) were normalized to GAPDH, and results are expressed as scatter dot plots including the median relative expression levels in each group. Symbols—○: for control1, □: for control2, Δ: for control3, ◊: for control4. Data were analyzed using paired *t*-test, and *p* values < 0.05 were considered significant.

We evaluated the effect of *MLLT11* knockdown on stromal cell adhesion and invasion. The MLLT11 knockdown did not significantly affect hESC invasion (Supplementary Figure S3a). Knockdown of *MLLT11* appeared to result in an increase in cellular adhesion at 10 min after plating on a collagen/fibronectin-coated plate (Figure 6a, left). Quantification confirmed that the number of adhered cells after 10 min was significantly increased in *MLLT11* knockdown cells (median change of 0.625, *p* = 0.001) (Figure 6a, middle). Further, we measured the area covered by adhered cells. This also revealed a significant increase in the area covered by adherent cells following *MLLT11* knockdown (median change of 0.070, *p* = 0.002 (Figure 6a, right). These data were consistent with the data obtained from the analysis of the differences in cellular adhesion in control and ectopic hESCs (Supplementary Figure S4). As for knockdown cells, the number of adhered cells in EcESM was increased (27.6% higher, *p* = 0.038) when compared to hESC controls. The area of adhered cells in EcESM was 35.4% higher (*p* = 0.017) compared to hESC controls (Supplementary Figure S4). 

The adhesion of endometrial stroma cells is required to establish ectopic lesions. A positive association between the levels of expression of MLLT11 and CD44, an adhesion molecule and direct target of the WNT/beta-catenin signaling, has been reported in breast cancer [17]. As reduced CD44 expression has been reported in the stroma cells of endometriosis lesions [26], we tested the effects of *MLLT11* knockdown on *CD44* expression and WNT pathway activity. The results from the RT-qPCR analysis of *CD44* (Supplementary Figure S3b) and Western blot analysis of active beta-catenin (Supplementary Figure S3c) in control siRNA and *MLLT11* siRNA-transfected hESCs show that these molecules are dispensable for the increase in adhesion seen in *MLLT11* knockdown cells. 

Another gene associated with the regulation of cellular adhesion is *ACTA2* [27,28]. *ACTA2* is upregulated in ectopic lesions compared to eutopic controls in our cohort, irrespective of endometriosis disease severity (Figure 6b). Therefore, we analyzed the effect of *MLLT11* knockdown on *ACTA2* expression. We found that the *MLLT11* knockdown caused a significant increase in ACTA2 at the mRNA (1.51 median fold increase; *p* = 0.008) and protein level (1.46 median fold increase; *p* = 0.023) compared to cells treated with control siRNA (Figure 6c). This effect was also associated with the upregulation of TGFB2 expression in *MLLT11* knockdown cells (1.63 median fold increase; *p* = 0.007) compared to siRNA-treated control cells (Figure 6d). 

As TGFB2 was previously reported to be a positive regulator of ACTA2 expression [29,30], we tested if this is also true in MLLT11 knockdown hESCs. We treated control siRNA and *MLLT11* siRNA-transfected cells with 5 µM of A83-01 TFGB2 inhibitor. Similar to untreated cells, as shown in Figure 6c, DMSO-treated cells showed an upregulation in *ACTA2* gene expression following *MLLT11* knockdown. However, this *ACTA2* upregulation was not reversed after TGFB2 inhibition for 24 h (Figure 6e), indicating that, in endometrial stromal, the two genes are regulated independently by MLLT11. 

## 3. Discussion

*MLLT11* is a gene implicated in hematopoietic progenitor cell differentiation [31] and neuronal cell differentiation [32]. In a pathogenic context, MLLT11 has been reported to play an oncogenic role in the development and progression of a variety of human cancers [13,14,15,16,17,18,19,20] and to act as a tumor suppressor in glioma neural carcinomas [21]. However, the role for MLLT11 in the pathogenesis of endometriosis has not been previously reported. In this study, we found that MLLT11 protein is significantly downregulated in the endometriosis lesion stromal cells of women with Stage III and IV endometriosis. Given the small number of samples of women with Stage I + II endometriosis in our study, we cannot exclude that MLLT11 is also downregulated in these patients. The knockdown of *MLLT11* in primary hESC of women without the disease leads to the downregulation of stromal cell proliferation, increased cell adhesion and reduced cell sensitivity to apoptotic stimuli. The effects of *MLLT11* knockdown on control hESC proliferation is due to disruption to the cell cycle, with G1 arrest and an impaired S phase associated with the upregulation of the cell cycle checkpoint proteins p21 and p27. Reduced proliferation endometriosis lesion stroma cells have been previously shown in vivo [9,33] and in vitro [34,35], and increased levels of p27 expression in endometriosis lesions have also been reported by several investigators [36,37]. Interestingly, Okamura et al. [36] showed that the levels of p27 increases with the progression of peritoneal endometriosis, with lower levels seen in highly vascularized and proliferating red peritoneal lesions compared to black lesions that show significantly lower proliferation based on the levels of Ki67 expression. Here, we show that reduced cell proliferation in MLLT11 knockdown primary stromal cells from controls resembles the behavior of primary endometriosis stroma cells, which express significantly lower levels of MLLT11 compared to controls. This indicates that MLLT11 can act as a regulator of endometriosis lesion stroma cell proliferation in vivo and could be an important factor in disease progression. The mechanism by which MLLT11 may regulate p21 and p27 protein expression in endometriosis remains unclear, but our data for p27 indicate that regulation occurs at a post-transcriptional level.

High levels of *MLLT11* were previously associated with low levels of *p21* expression and increased proliferation in bladder cancer cells, where the levels of *MLLT11* have been shown to be epigenetically regulated by miR-411 [19]. Mir-411 was identified as significantly upregulated in tissues of women with ovarian and peritoneal endometriosis compared to controls [38]. This indicates that a mechanism for the epigenetic regulation of *MLLT11* and *p21* expression, similar to those in bladder cancer, may exist in endometriosis. However, the discrete mechanisms of MLLT11 and MLLT11-mediated p21 regulation in endometriosis lesions remain to be experimentally tested. 

In the context of hepatocellular, ovarian and squamous carcinoma and in promyelocytic leukemia cells, MLLT11 was shown to exert proapoptotic functions [16,25,39]. However, the loss of MLLT11 in developing cortical neurons had no impact on cell death [22]. In our cell system, CTP induces apoptosis through the transcriptional activation of *c-MYC*, leading to the downregulation of *BCL2* and the *BCL2/BAX* ratio. In general, the ability of c-MYC to induce apoptosis has been reported [40]. Previously, we could show a significant downregulation of c-MYC protein expression in the stromal compartment of ectopic endometriosis lesions, suggesting that cells with reduced c-MYC are more prone to survive apoptotic stimuli [41]. However, our data showed that *c-MYC* expression is not under the control of MLLT11. The knockdown of *MLLT11* in primary control hESCs did not affect the basal apoptosis levels or the *c-MYC* expression levels but showed a significant effect on the survival of CPT-treated cells. This was associated with changes in the degree of BCL2 gene expression and the *BCL2*-to-*BAX* ratio in knockdown CPT-treated cells, leading to the enhanced antiapoptotic property of the cells. 

CPT treatment can induce oxidative stress by enhancing the accumulation of intracellular reactive oxygen species (ROS) [42], leading to lipid peroxidation and lipid-derived electrophile accumulation [43], which induces cellular damage [44], normally leading to apoptosis. Oxidative stress is known as one of the main factors in endometriosis development and progression [45], and a decreased sensitivity of endometriosis lesion stroma cells to oxidative stress inducers has already been shown [46].

As CPT is a DNA topoisomerase 1 inhibitor, whose activity is higher in proliferating cells [43], the increased survival of control hESCs with reduced MLLT11 expression to oxidative stress inducers, such as CTP, might be due to reduced proliferation and cell cycle arrest, accompanied by changes in the regulation of the BAX and BCL2 signaling. Our data show that MLLT11 might be an important factor in the regulation of cell survival in response to oxidative stress. 

Furthermore, adhesion of endometrial tissue fragments to pelvic mesothelium is required for the formation of ectopic lesions. In our study, we demonstrated that MLLT11 is a negative regulator of hESC adhesion. The increased adhesion following *MLLT11* knockdown in hESCs was accompanied by the significant transcriptional activation of *TGFB2* and *ACTA2* genes. Increased expression of TGFB2 was found in ectopic lesions of women with endometriosis [47,48], and in rat models, high levels of TGFB2 were associated with more advanced stages of the disease [47]. TGFB2, as positive regulator of ACTA2 expression, was previously reported in bovine retinal pigment epithelial cells [30]. We have shown that inhibition of TGFB2 signaling does not affect the expression of ACTA2 in both control siRNA and MLLT11 siRNA-transfected cells. Therefore, in endometriosis stroma cells, the MLLT11 regulates *TGFB2* and *ACTA2* expression independently, and TGFB2 signaling does not seem to be involved in the regulation of ACTA2. An increased level of ACTA2 expression within ectopic endometriosis lesions compared to endometrium tissues from women without [49,50] and with endometriosis [37,49] was reported. In baboons, the intrastromal ACTA2-positive cells increased as the endometriotic lesions progressed [51], suggesting that ACTA2 is associated with more advanced stages of the disease. We confirmed this observation and showed that ACTA2 expression is more prominent in the endometriosis lesions of women with more advanced endometriosis. 

We have shown that primary ectopic stroma cells of women with endometriosis, expressing lower levels of MLLT1 and higher levels of ACTA2, compared to primary cells of women without the disease, displayed increased adhesive properties. Given that in vitro inhibition of ACTA2 in myofibroblast cells leads to a significant decrease in adhesion due to the reduction in the focal adhesion maturation [27], we speculate that the increased ACTA2 expression in *MLLT11* knockdown cells might increase the adhesive properties in these cells. Myofibroblasts are the main source of the extracellular matrix in fibrosis, and the myofibroblast differentiation of endometriosis stroma cells includes an activation of ACTA2 expression [52]. Therefore, the findings in the present study suggest that reduced *MLLT11* expression in endometriosis stroma cells may regulate the profibrotic capacity of these cells and contribute to endometriosis-associated fibrosis. However, additional experimental work needs to be performed to investigate the discrete mechanism of ACTA2 regulation and the role of ACTA2 in endometriosis stromal cell adhesion. A limitation of this study are the relative low numbers of patient samples, in particular from patients with mild endometriosis (rAF I + II), that were used.

Overall, our findings are consistent with a model in which a decreased expression of MLLT11 promotes the persistence of endometriosis lesions outside of the uterus by increasing cell adhesion and enhancing the resistance of endometriosis stroma cells to oxidative-stress-mediated apoptosis. The low levels of expression of MLLT11 in the stroma cells of endometriosis lesions are also consistent with the lack of uncontrolled proliferation of the lesions, ensuring the benign nature of the disease. However, whether the levels of MLLT11 expression could correlate with transformation of the lesion from benign (low MLLT11) to a malignant (high MLLT11) phenotype, particularly in endometriosis-associated ovarian cancer, needs further investigation.

Our results indicate that MLLT11 may be a new clinically relevant player in the pathogenesis of endometriosis, in particular in the advanced stages of the disease.

## 4. Materials and Methods

### 4.1. Study Population

Tissue was collected following the procedures of the Endometriosis Marker Austria (EMMA) study, a prospective study at the Tertiary Endometriosis Referral Center of the Medical University of Vienna. Premenopausal women aged 18–50 years that were given laparoscopic surgery due to suspected endometriosis, chronic pelvic pain, infertility, uterine leiomyoma or benign adnexal masses were invited to participate in the EMMA study. Ethics approval for this study was obtained from the Medical University of Vienna institutional ethics committee (EK 545/2010). Written and verbal informed consent from each patient was obtained before inclusion in the study. Detailed patient characteristics are summarized in Supplementary Table S1A–C. From the 70 women that participated in the study, 33 were endometriosis-free controls, and 37 were endometriosis patients. Endometriosis disease severity was defined by the revised American Fertility Society (rAFS) score. The control group were women subject to laparoscopic surgery due to uterine fibroids, fallopian tube disorders and benign ovarian cysts or due to unexplained chronic pelvic pain or infertility. Pregnant women and those breastfeeding within the last 6 months, as well as those who had received hormonal treatment within the last 3 months, were excluded from the study. Additionally excluded were women with known or suspected infectious disease, with acute inflammation and those with chronic autoimmune or malignant disease. Endometriosis was confirmed macroscopically by qualified surgeons and postsurgery through histological analysis by a pathologist. No endometriosis was detected in control patients at the time of the laparoscopic surgery. Control patients contributed one eutopic endometrium sample, while some endometriosis patients contributed multiple endometriosis lesion samples. All tissue samples were collected during diagnosis and/or therapy of endometriosis via curettage or laparoscopic surgical intervention. Samples were collected according to the Endometriosis Phenome and Biobanking Harmonization Project guidelines [53].

### 4.2. RNA Isolation

Frozen tissue samples taken from 31 control patients and 23 endometriosis patients were homogenized with a Precellys 24 homogenizer (Supplementary Table S1A, PEQLAB, Erlangen, Germany). Total RNA was then isolated from eutopic and ectopic endometrium samples using the Agilent Absolutely Total RNA kit, which includes DNaseI treatment (Agilent, Santa Clara, CA, USA). Total RNA was isolated from primary stromal cells using the RNeasy mini kit (Qiagen, Venlo, The Netherlands) and then treated with DNAseI using the RapidOut DNA removal Kit (Thermo Fisher Scientific, Waltham, MA, USA). RNA concentration and purity were then measured using a NanoDrop ND-1000 spectrophotometer (NanoDrop Technologies, Wilmington, DE, USA). We defined the RNA quality as sufficient when the OD260/280 and OD260/230 ratios were around 2.00.

### 4.3. Quantitative Reverse Transcription PCR (RT-qPCR)

RNA was reverse transcribed with the SuperScript^®^ III First-Strand Synthesis Reverse Transcriptase kit using a mixture of oligo-d (T) and random hexamer primers (Life Sciences Advance Technology, St. Petersburg, FL, USA). We then diluted the cDNA 2-fold with water before assaying gene expression with quantitative reverse transcription PCR (RT-qPCR). Each RT-qPCR reaction contained 1× TaqMan master mix (Applied Biosystems, Waltham, MA, USA with ROX reference dye) and one of the gene expression TaqMan Assays listed in Supplementary Table S2A. RT-qPCR was conducted using a 7500 Fast Real-Time PCR System (Applied Biosystems, Waltham, MA, USA), with an initial denaturation step for 10 min at 95 °C, followed by 40 cycles of 15 s at 95 °C and 1 min at 60 °C. Relative expression of target genes was calculated using either the -deltadelta-CT method for in vitro analysis as described [54] or using the -delta-CT method for patient data analysis as described [55], with normalization to GAPDH or ACTB expression, as indicated in the figure legends.

### 4.4. Immunohistochemistry

MLLT11 protein expression and cellular localization was detected using immunohistochemical (IHC) analyses of archival formalin-fixed, paraffin-embedded tissue samples from control (n = 24) and endometriosis cases (n = 19) collected in the pathology department of the Medical University of Vienna between 2012 and 2015 (Supplementary Table S1B). IHC staining was performed on three-micrometer-thick tissue sections following heat antigen retrieval with 10 mM sodium citrate buffer (pH = 6) and blocking with H_2_O_2_ on Ultra V Block (Thermo Scientific, Ultra Vision LP Kit, TL-060-HL, Waltham, MA, USA). 

The CD10 antibody (mouse IgG, NCL-CD10-270, Novocastra, Wetzlar, Germany) and the MLLT11 antibody (rabbit IgG, ab109016, Abcam, Cambridge, GB) were applied with antibody diluent and background reducing components buffer (Dako, S3022, Glostrup, Denmark). Kidney was used as a positive control for MLLT11 and placenta for CD10. Mouse IgG1 (760-2014, Ventana, CA, USA) and rabbit IgG (sc-2027, Santa Cruz, CA, USA) were used as isotype negative controls. The IHC signals were detected using Ultra Vision LP Kit after incubating the enhancer for 10 min and the HRP polymer for 15 min (Thermo Scientific, Ultra Vision LP Kit, TL-060-HL, MA, USA) and then further incubation with DAB-Substrate (Dako, K346811, Glostrup, Denmark) according to the manufacturer’s protocol. Slides were counterstained in hematoxylin before they were dehydrated and mounted. Scoring and immunohistochemical analysis was performed, as described previously [56] and as outlined in the figure legends. 

### 4.5. Primary Endometrial Stromal Cell (hESC) Cultures

Endometrial tissue obtained from control patients by curettage or from ectopic lesions from endometriosis patients by laparoscopic surgery was used to make primary endometrial stromal cells as previously described [57]. Briefly, the tissue was minced and incubated with collagenase (Sigma-Aldrich, St. Louis, MO, USA) at 37 °C for 10 min, filtered and then cultured further, as previously documented [58]. The cells were cultured on fibronectin–collagen-(Gibco, Grand Island, NY, USA) coated dishes in DMEM-F12 without phenol red (Gibco) supplemented with 10% fetal bovine serum (FBS) (Gibco), 2 mM L-glutamine (Gibco) and 1% antibiotics–antimycotic (Gibco) up until a maximum of passage 7 in a 37 °C CO_2_-humified incubator. The purity of these stromal cells was evaluated using immunofluorescence analysis with antibodies against vimentin (stromal cell marker), CD10 (endometrial stromal cell marker) and by qPCR using PECAM1 (endothelial marker) and EPCAM (epithelial marker). This method produces 95–99% pure stromal cells.

### 4.6. Immunofluorescence

Next, 2 × 10^4^ primary cells were plated on fibronectin/collagen (Roche, Mannheim, Germany) and allowed to adhere overnight. Briefly, the adhered cells were fixed in 4% paraformaldehyde, incubated in 50 mM ammoniumchloride, permeabilized and blocked in 0.3% Triton-X-100 and PBS with 5% normal goat serum (5425) before incubation with the primary antibodies: vimentin (rabbit IgG, sc5565; Santa Cruz Biotechnology, Santa Cruz, CA, USA) and CD10 (mouse IgG, NCL-CD10-270, Novocastra). Antirabbit Alexa Fluor 488-conjugated phalloidin (A1494754, Invitrogen, Waltham, MA, USA) and antimouse (A11017, Invitrogen) were used to visualize vimentin (rabbit) and CD10 (mouse) staining, respectively. DAPI was used for nuclear staining (10236, Roche Diagnostics, Indianapolis, IN, USA). Rabbit IgG (sc-2027, Santa Cruz) and mouse IgG1 (760-2014, Ventana) were used as isotype controls. Epifluorescence imaging was performed using an Olympus BX50 microscope equipped with soft imaging system-F-View camera and Cell^P imaging software V2.1 (Olympus Austria Ges.m.b.H, Vienna, Austria).

### 4.7. MLLT11 Knockdown

The primary hESCs were cultured in complete culture cell medium on coated 6-well culture plates (Nunc, Thermo Fisher Scientific, Waltham, MA, USA) at a concentration of 8 × 10^4^ cells/well until 50% confluency. The cells were then transfected with a MLLT11 targeting siRNA oligo or a nontargeting control siRNA oligo (Cat. 4390846, Ambion, Austin, TX, USA) at a final concentration of 10 nM using Lipofectamine RNAiMAX transfection reagent (Invitrogen by Life Technology, Waltham, MA, USA). Western blot analyses and phenotypic analysis of *MLLT11* knockdown cells for changes in cellular proliferation and invasion/adhesion were conducted 72 h post-transfection. RT-qPCR to assess relative RNA expression levels was conducted 48 h post-transfection.

### 4.8. Protein Isolation and Western Blot

Cells were lysed in a whole-cell lysis buffer containing 1% Triton-X 100, 10 mM Tris-HCl, pH7.4, 150 mM NaCl and 5 mM EDTA. Phosphatase and a protease inhibitor cocktail (phosStop, cOmplete mini, EDTA free, Thermo Fisher Scientific, Waltham, MA, USA) were added to the lysis buffer immediately prior to use. A standard Bradford assay was used to determine the protein concentration. An equal amount of protein from each sample was then immunoblotted and incubated with primary antibodies for the proteins of interest (Supplementary Table S2B) overnight at 4 °C. Secondary antibodies were diluted in a Tris pH 8.0, 0.1% Tween 20 buffer and incubated with the sample for 1 h at room temperature. Bound antibodies were then detected with the horseradish peroxidase chemiluminescent Clarity Western ECL substrate (Bio-Rad Laboratories Inc., Hercules, CA, USA) and visualized on a ChemiDoc Imaging System (Bio-Rad Laboratories Inc., Hercules, CA, USA). Protein expression levels on the blot were then quantified using ImageJ Software V1.8.0 accessed on 2 July 2023 (http://rsbweb.nih.gov/ij).

### 4.9. Flow Cytometry

We used standard propidium iodide (PI) DNA staining flow cytometry protocol to detect changes in the cell cycle following *MLLT11* knockdown, Briefly, per assay, we harvested 1 × 10^6^ cells 72 h post-siRNA knockdown and fixed them in 70% precooled ethanol for 2 h on ice. Following a PBS wash, the cells were resuspended in 10 µL PI staining solution containing 0.5 mL PI/RNAse (550825 and 51-6621-1E, BD Pharmingen™, Heidelberg, Germany) and incubated at room temperature for 10 min. PI positive cells were then quantified using flow cytometry. The effect of MLLT11 knockdown on apoptosis was assessed using flow cytometry, following staining with an FITC-conjugated AnnexinV antibody (antibody 640906, Annexin V Binding Buffer, (422201), BioLegend, San Diego, CA, USA) and the vital dye 7-amino-actomycin D (00-6993-50, eBioscience, San Diego, CA, USA). A total of 10,000 events were recorded for each sample, with unstained cells being used as an assay control.

### 4.10. Proliferation Assay

The proliferation rate 72 h after *MLLT11* knockdown in primary endometrial stromal cells derived from control patients and from ectopic lesions from endometriosis patients was analyzed using the CyQuant direct cell proliferation assay (Invitrogen, Waltham, MA, USA). Cells were trypsinized 48 h post-transfection with MLLT11 and control siRNA oligos, and 15,000 cells/well were seeded on 96 flat-bottom cell culture plates (Thermo Fisher Scientific, Roskilde, Denmark). After 12 h, the cells were stained for 1 h with 100 µL CyQuant reaction, and then, fluorescence was measured on a Clariostarplus microplate reader fitted with appropriate filters (BMG Labtech, Ortenberg, Germany). Following correction for background fluorescence with a cell-free sample, a standard curve was calculated and used to estimate cell number. Experiments were performed in triplicate, and the mean number of proliferating cells plotted relative to control siRNA-treated cells and set to 1.

### 4.11. Matrigel Invasion Assay

Invasion of cells through a matrigel barrier was detected with a Boyden chamber assay 48 h post-siRNA knockdown. For each assay, 15,000 cells were resuspended in 100 µL media and added to a Matrigel-coated filter (Matrigel growth factor reduced (354,230); Corning Incorporated, Corning, NY, USA, 1% matrigel solution in PBS, filter: 6.5 mm diameter, 8 m pores, Corning Incorporated, Corning, NY, USA). Cells were left to invade for 12 h toward the bottom of the well containing media with 10% FCS. Cells on the lower surface of the filter were fixed with 4% PFA and stained with DAPI and were photographed with 10× objective using the EVOS2000 microscope. The mean number of invaded cells was calculated over four independent fields, and the results of triplicate experiments presented. Cell counting was conducted using ImageJ software V1.8.0 accessed on 2 July 2023 (http://rsbweb.nih.gov/ij).

### 4.12. Adhesion Assay

The ability of cells to adhere on fibronectin- and collagen-coated 24-well plates was measured. After 72 h of siRNA knockdown, 30,000 cells per assay were resuspended in 500 µL of growth media supplemented with 10% *v*/*v* FCS and antibiotics. The cells were allowed to attach for 10 min or for 30 min and then fixed with ice cold methanol and stained with Chrystal Violet. Photos were taken using a EVOS2000 microscope (Thermo Fisher Scientific, Waltham, MA, USA) with an ×10 objective. The number of adhering cells per experiment was calculated by averaging the number of attached cells over four independent fields, with the results of triplicate experiments presented. Adhesion areas were calculated using the average number of pixels for each cell in a photographic field over four independent fields per experiment and expressed as averages of triplicate experiments. ImageJ software V1.8.0 accessed on 2 July 2023 was used for counting the number and the area of adhering cells (pixels2) accessed on 2 July 2023 (http://rsbweb.nih.gov/ij).

### 4.13. Statistics

All statistical tests were performed using Prism (GraphPad Prism 9.0 software, La Jolla, CA, USA). The exact statistical procedures for each analysis are described in the corresponding figure legends.

## Data Availability

Data are contained within the article or Supplementary Materials.

## Supplementary Materials

The following supporting information can be downloaded at: https://www.mdpi.com/article/10.3390/ijms25010439/s1.
